# Galectin-10, a Potential Biomarker of Eosinophilic Airway Inflammation

**DOI:** 10.1371/journal.pone.0042549

**Published:** 2012-08-06

**Authors:** Justin C. Chua, Jo A. Douglass, Andrew Gillman, Robyn E. O'Hehir, Els N. Meeusen

**Affiliations:** 1 Department of Allergy, Immunology and Respiratory Medicine, The Alfred Hospital and Monash University, Melbourne, Victoria, Australia; 2 Biotechnology Research Laboratories, School of Biomedical Sciences, Monash University, Clayton, Victoria, Australia; Instituto de Biofisica Carlos Chagas Filho, Universidade Federal do Rio de Janeiro, Brazil

## Abstract

Measurement of eosinophilic airway inflammation can assist in the diagnosis of allergic asthma and in the management of exacerbations, however its clinical implementation remains difficult. Galectin-10 has been associated with eosinophilic inflammation and has the potential to be used as a surrogate biomarker. This study aimed to assess the relationship between galectin-10 in sputum with sputum eosinophil counts, the current gold standard of eosinophil inflammation in the lung. Thirty-eight sputum samples were processed for both eosinophil counts by cytospins and semi-quantitative measurements of galectin-10 by western blots. A strong association was observed between galectin-10 levels in sputum and sputum eosinophil measurements, and they accurately determined sputum eosinophilia. The results support the potential for galectin-10 to be used as a surrogate biomarker of eosinophilic airway inflammation.

## Introduction

Asthma is a common condition, affecting 300 million people worldwide and causing significant morbidity, mortality and economic loss [1]. It is a chronic inflammatory disorder of the airways, with inflammatory cells and mediators consistently implicated in the disease process [2], [3], [4].

Recently, asthma has been considered a heterogeneous disease with various inflammatory subtypes, particularly eosinophilic inflammation [5]. The presence of eosinophilic inflammation in asthmatic disease is classified by the proportion of these cells in sputum, with the broad classification of eosinophilic (≥3%) or non-eosinophilic asthma (<3%) [6]. Several studies have demonstrated that these subtypes are reproducible and remain consistent over time, and there is growing evidence that eosinophilic inflammation is associated with exacerbations and improved responsiveness to corticosteroids compared to non-eosinophilic inflammatory subtypes [7], [8], [9].

Current asthma assessments, such as lung function and airway hyper-responsiveness poorly predict inflammatory subtypes of asthma [7], [8], [10], [11]. Objective, non-invasive tests that focus on the inflammatory component of asthma have been devised, with the most researched methods being sputum eosinophil percentages and fractional exhaled nitric oxide (FE_NO_). A perceived advantage is that they provide a more direct and sensitive assessment of airway inflammation [12], [13]. A number of randomized controlled trials have demonstrated that the titration of corticosteroid medications according to sputum eosinophil percentage is protective for asthma exacerbations when compared to traditional asthma assessments (symptoms and spirometry) [2], [10]. However, the applicability of sputum eosinophil counts has been limited by the time-consuming nature of sputum induction and analysis [13]. By contrast, FE_NO_ is simple to measure; however, the evidence of its potential benefits has been mixed [7], [14].

Considering the clinical relevance of sputum eosinophils, biomarkers of eosinophilic airway inflammation that are more amenable to the development of automated or rapid point-of-care assays are needed [15], [16]. Galectin-10 is a major constituent of human eosinophils, also known as the Charcot-Leyden crystal protein (CLCP) [17], [18]. This glycan binding protein characteristically forms bi-pyramidal hexagonal crystals, known as the Charcot-Leyden crystals, which have long-been linked with eosinophilic inflammation [17], [19] Though the function of galectin-10 remains largely unknown, several studies have clearly demonstrated its association with eosinophilic inflammatory diseases such as asthma and allergic rhinitis [20], [21], [22], [23], [24]. An ovine orthologue, galectin-14, has been shown to be secreted in the airway mucus of a sheep asthma model closely associated with lung eosinophilia [18], [25]. In addition, galectins have been increasingly recognized as potential biomarkers for several disease processes [26].

To date, there have been no studies comparing the quantity of galectin-10 in sputum to sputum eosinophil percentages, the gold standard measurement of eosinophilic airway inflammation in airway diseases. If galectin-10 is found to have a strong link to eosinophilic airway inflammation, then it could be developed into a rapid diagnostic assay and provide an assessment tool with the same clinical benefits as sputum eosinophil percentages, but with improved clinical applicability.

## Methods

### Subjects

Participants were recruited from the Alfred Hospital clinics, and included patients with asthma and allergic bronchopulmonary aspergillosis (ABPA), asthma, bronchiectasis and interstitial pulmonary fibrosis (IPF) and healthy controls (Table 1).

**Table 1:** Clinical Characteristics of Participants.

Participant Diagnosis Groups	Asthma with ABPA(n = 7)	Asthma without ABPA(n = 10)	Healthy control(n = 7)	Bronchiectasis(n = 3)	IPF(n = 1)
Mean age (yrs)	59.3	39.3	26.6	47.3	56.0
Gender (female)	5	4	4	3	0
Pre-BD FEV_1_(% predicted)	60.0(48.5–70.8)	76.5(63.0–89.0)	99.0(91.5–102)	57.0(49.5–63.0)	59.0
Post-BD FEV_1_ change (%)	5.0(3.0–7.0)	12.0(6.0–14.0)	2.0(1.5–3.5)		
Daily oral corticosteroid dose	10.0(1.5–18.75)	0.0(0.0–10.0)	0.0(0.0–0.0)	0.0(0.0–0.0)	0.0
Daily inhaled corticosteroid dose (mcg of budesonide equivalent)	1600(900–2000)	450(0–2000)	0.0(0.0–0.0)	800(400–1400)	
Daily salbutamol requirements (number of puffs, 100 mcg dose)	6.0(1.0–7.0)	0.5(0.0–2.5)	0.0(0.0–0.0)	0.5(0.25–2.25)	0.0

BD FEV_1_ = bronchodilator forced-expiratory-volume-in-1-second.

Informed, written consent was obtained from all participants, and medical history and spirometry values were recorded [27], [28]. The study was approved by the Alfred Hospital and Monash University Human Research Ethics Committees (approval number 39/10).

### Sample Acquisition and Sputum Eosinophil Percentages

Participants were asked to expectorate a spontaneous sputum sample or, if unable to do so, they underwent sputum induction with the inhalation of hypertonic saline (4.5%) through an ultrasonic nebulizer (Suchatzki, Rennerod, Germany). Samples were kept on ice and processed within 2-hours according to the method of Pavord et al.,1997 [29]. Briefly, sputum was selected, weighed and placed in four-times the volume of dithiothreitol (DTT) 0.1% (Sputolysin Reagent, Calbiochem, New Jersey, USA), agitated for 15 minutes on ice and an equal volume of PBS added. The suspension was filtered through a 70 µm cell filter, viable cells counted, and centrifuged at 4°C, 1200 rpm, for 10 minutes. The supernatant was stored at −70°C for future galectin-10 analysis. Cells were resuspended in PBS at 0.75×10^6^ cells/ml and cytospins stained with Diff-Quik. A minimum of 300 cells were counted. A sputum sample was considered positive for eosinophilic airway inflammation if eosinophils were ≥3% [5], [8], [9]. If >50% of cells were squamous cells, the sample was excluded from analysis.

### Galectin-10 Measurement using Western Blots

Reducing sample-buffer was added to the sputum supernatant in a 1∶1 dilution, run on an SDS-PAGE gel and transferred to nitrocellulose membranes. Membranes were incubated with 1.0 µg/mL anti-human galectin-10 goat IgG-antibody (R&D systems, Minnesota, USA), followed by anti-sheep Ig-horseradish peroxidase (HRP) antibody (DAKO, Glostrup, Denmark) at a concentration of 1/8000. The reaction was detected by Enhanced Chemiluminescence (ECL) (GE Healthcare, Buckinghamshire, UK), and the optical density of the bands was measured using ImageJ (Rasband, National Institutes of Health).

**Figure 1:**
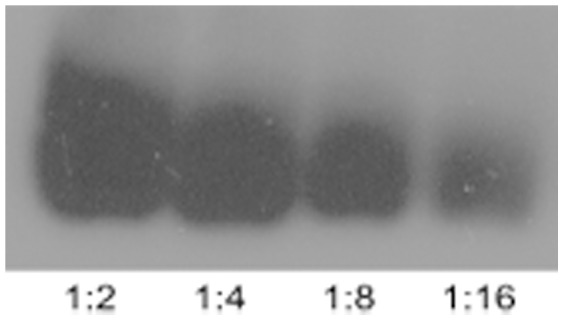
A Western blot demonstrating the positive control sample diluted to varying concentrations to create a calibration curve. The numbers at the bottom of the bands represent the degree of dilution (initial sample volume: total diluted volume).

To semi-quantify the samples, a sputum sample from a participant with high levels of galectin-10 was chosen as a positive control in all western blot assays (Figure 1). Different sample bands' optical density were calculated as a percentage of the positive control band multiplied by a factor of 100 and named the Relative Galectin-10 Unit (RGU).

The protein concentrations of sputum samples were measured using the Bradford protein assay.

### Statistical Analysis

A Kruskal-Wallis test was performed with post-hoc comparisons by a Mann-Whitney U test to determine significant differences between groups. The interquartile range (IQR) was calculated as a measure of dispersion. Significant p-values were adjusted to <0.01 due to the higher chance of type-1 errors. Correlations were evaluated by Spearman's correlation co-efficient. A receiver-operating-characteristic (ROC) analysis was performed to determine the accuracy of sputum galectin-10 in determining sputum eosinophil percentage results ≥3%. All data were analyzed with SPSS for Windows (version 17.0).

## Results

### Recovery and protein concentration of sputum samples

In total, 38 analyzable sputum samples were obtained from 28 patients, consisting of 16 asthma with ABPA, 11 asthma without ABPA, 7 healthy controls, 3 bronchiectasis, and 1 IPF samples (Table 1). Of these, sputum induction was performed to obtain 14 samples, 5 from patients who had asthma without ABPA and 9 from healthy controls. Out of these samples, one resulted in squamous cell contamination while another was unsuccessful in inducing an adequate sample, resulting in a success rate of 85.7%. The induced sputum samples had a median protein concentration of 0.346 mg/mL (IQR: 0.316–0.406) which was significantly lower than the median of the spontaneously produced sputum samples of 0.929 mg/mL (IQR: 0.668–1.204).

The asthmatic samples (asthma with or without ABPA) had a significantly greater median protein concentration compared to the healthy control samples (p<0.001), with concentrations of 0.843 mg/mL (IQR: 0.535–1.086) and 0.355 mg/mL (IQR: 0.330–0.391) respectively.

### Sputum cell counts

**Figure 2:**
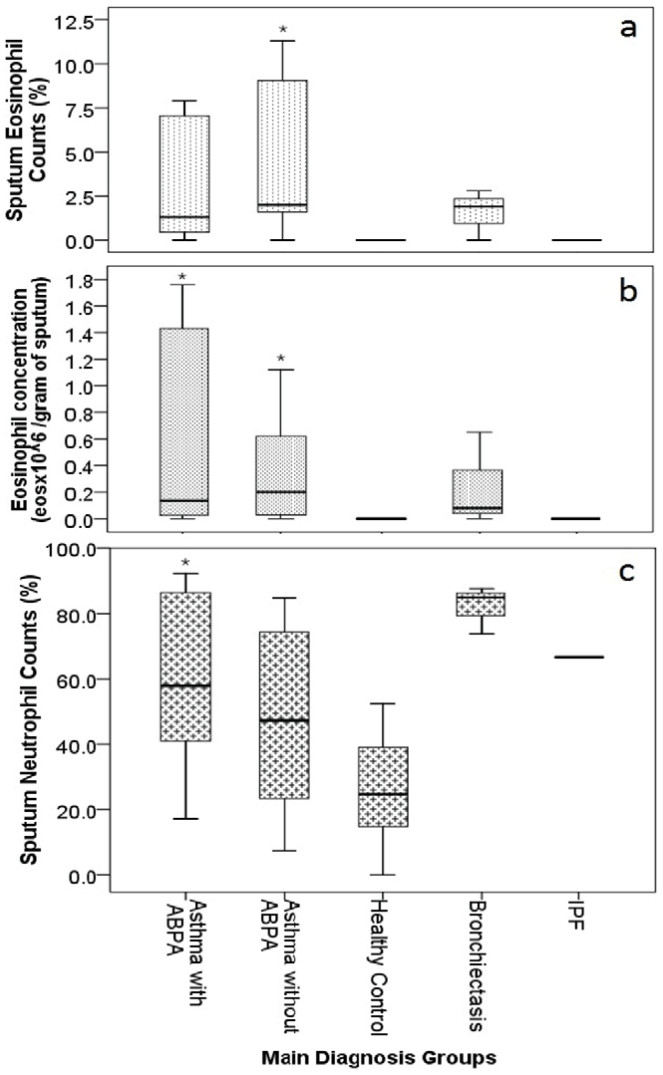
Sputum eosinophil (a&b) and neutrophil (c) levels across the main diagnosis groups. * = significant difference with the healthy control group (p<0.01).

All differential cell counts of the sputum samples are shown in Table 2 and Figure 2. Out of 38 analyzable samples, 11 had >3% eosinophils. The majority of the positive counts came from the asthma with ABPA group with 7 positive results and the remaining 4 from the asthma without ABPA group. By this criterion, there were no positive sputum eosinophil counts among the healthy control, bronchiectasis and interstitial pulmonary fibrosis participants.

**Table 2:** Medians (interquartile ranges) of cell counts and concentrations across the main diagnosis groups.

Participant Diagnosis Groups	Asthma with ABPA(n = 7)	Asthma without ABPA(n = 10)	Healthy control(n = 7)	Bronchiectasis(n = 3)	IPF(n = 1)
Positive counts (>3% eosinophils)	7	4	0	0	0
Sputum eosinophil count (%)	1.3(0.5–7.1)	2.0*(1.6–9.1)	0.0(0.0–0.0)	1.9(1.0–2.4)	0.0
Eosinophil sputum concentration(×10^6^ eosinophils/gram of sputum)	0.14*(0.03–1.43)	0.20*(0.03–0.62)	0.00(0.00–0.00)	0.08(0.04–0.37)	0.00
Sputum neutrophil count (%)	57.9*(41.0–86.5)	47.3(23.4–74.4)	24.7(14.8–39.1)	85.0(79.4–86.3)	66.7

* significant difference with the healthy control group (p<0.01).

The asthma without ABPA participants had the highest median sputum eosinophil counts (Table 2, 2.0%), followed by the bronchiectasis participants (1.9%) and the asthma with ABPA participants (1.3%). The asthmatic samples had significantly higher sputum eosinophil counts than the healthy control group (p = 0.003), which had an absence of eosinophils. Both the asthmatic groups (with and without ABPA) reported large interquartile ranges, reflecting the variation in eosinophil percentages.

Sputum eosinophilia was also calculated as the total number of eosinophils per sputum weight (Table 2, Figure 2b). Similar to the sputum eosinophil counts, the asthma without ABPA participants had the highest median sputum eosinophil concentration of 0.20×10^6^ eosinophils per gram of sputum. However, the asthma with ABPA had a higher concentration than the bronchiectasis group (0.14×10^6^ vs. 0.08×10^6^ eosinophils per gram respectively). The asthmatic samples had a significantly greater eosinophil concentration than the healthy control group (p<0.001).

The bronchiectasis group was found to have the highest median sputum neutrophil count of 85.0% (Table 2). There was a significant difference in neutrophil counts between the asthmatics and the healthy control group (Figure 2c).

### Measurement of galectin-10 in sputum

**Figure 3:**
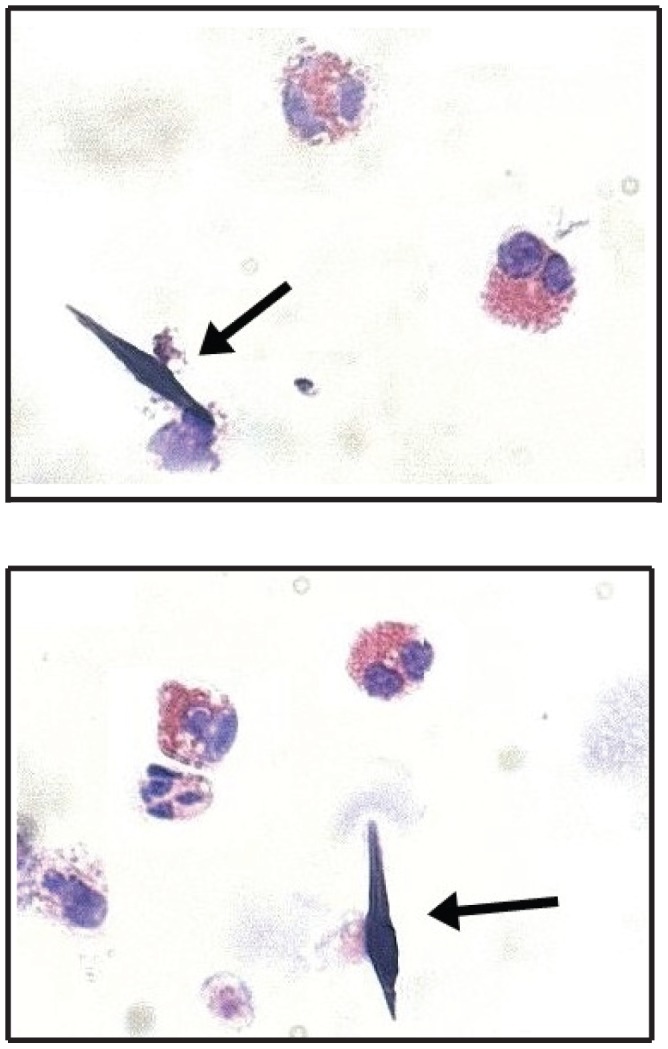
Cytospins of sputum samples from an asthma with ABPA patient which contained bi-pyramidal Charcot-Leyden crystals (arrows).

During microscopic examination of sputum cytospins, four samples with a high percentage of eosinophils (10.5%) were identified as containing the characteristic bi-pyramidal, hexagonal Charcot-Leyden crystals (Figure 3).

Results of the galectin-10 measurements of sputum supernatant across the diagnosis groups, relative to a standard positive sample, are shown in Table 3. Similar to the eosinophil counts, the galectin-10 levels within the two asthmatic groups were highly variable with large interquartile ranges. The asthma without ABPA group had the highest median galectin-10 amount (13.8 RGU), while the healthy control group and the IPF participant did not have any measurable galectin-10 in their sputum. The ABPA group was found to have a greater mean amount of galectin-10 than the bronchiectasis group (12.9 vs. 10.5 RGU respectively). The asthmatic samples had significantly more galectin-10 than the healthy control group (p<0.001), however the bronchiectasis group was not significantly different from control (Table 3).

**Table 3:** Median and interquartile ranges of the sputum galectin-10 amounts (RGU) across the main diagnosis groups.

Participant Diagnosis Groups	N	Median (IQR) of sputum galectin-10 amounts (RGU)	Significance of difference with healthy control group (p-value)
Asthma with ABPA	7	12.9 (7.9–52.5)	<0.001*
Asthma without ABPA	10	13.8 (3.3–22.5)	0.008*
Asthma (with or without ABPA)	17	13.1 (7.4–23.9)	<0.001*
Bronchiectasis	3	10.5 (5.3–15.3)	0.117
IPF	1	0.0	N/A
Healthy control	7	0.0 (0.0–0.0)	N/A

RGU = Relative galectin-10 units.

* <0.01 = significant value.

The amount of sputum galectin-10 was found to have a significant, moderate correlation with the protein concentration of the samples (r_s_ = 0.568, p<0.001).

### Correlation between sputum galectin-10 and sputum cell counts

**Figure 4:**
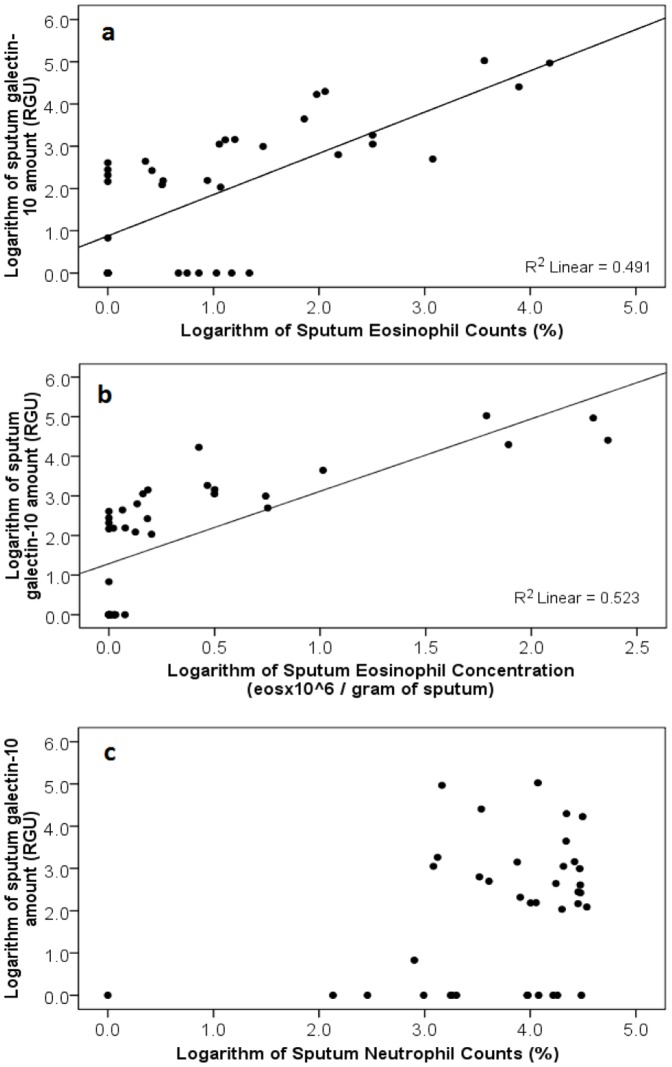
Correlations of sputum galectin-10 amount versus: (a) % sputum eosinophil count (rs = 0.695, p<0.001), (b): eosinophil concentration (rs = 0.786, p<0.001), and (c): % sputum neutrophil count (rs = 0.243, p = 0.141).

The sputum galectin-10 levels showed a highly significant correlation with sputum eosinophil counts (r_s_ = 0.695, p<0.001) (Figure 4a). The correlation was stronger with sputum eosinophil concentrations (r_s_ = 0.786, p<0.001) (Figure 4b). These correlations remained high and significant whether sputum samples were analyzed individually or results were averaged for each person (data not shown). When only the asthmatic samples were selected, the correlation of galectin-10 levels with sputum eosinophil counts and concentrations was even stronger, r_s_ = 0.722 (p<0.001) and r_s_ = 0.800 (p<0.001) respectively.

The sputum galectin-10 amount did not have a significant correlation with sputum neutrophil counts (r_s_ = 0.243, p = 0.141) (Figure 4c).

The receiver operating characteristic curve (ROC curve) for galectin-10 to identify a positive sputum eosinophil count had an area under the curve of 0.963 (p<0.001, 90% CI: 0.906–1.000), with a sputum galectin-10 threshold of 13.5 RGU resulting in a sensitivity of 100.0% and a specificity of 88.9%.

## Discussion

This study has demonstrated that galectin-10 in sputum has a strong correlation with sputum eosinophil percentages, and an even stronger correlation with sputum eosinophil concentrations. Our study also found that sputum galectin-10 had a high level of diagnostic accuracy to identify sputum eosinophil percentages ≥3%, the threshold most commonly used to guide treatment in asthma [5], [8], [9].

This is the first study to compare the amounts of galectin-10 in sputum with sputum eosinophil percentages, which is currently held as the gold-standard measurement of eosinophilic airway inflammation [8], [30]. There has been one past study which has compared sputum CLCP amounts in asthmatics with other respiratory diseases [23]. This study by Dor *et al* found that elevated CLCP levels were associated with both asthma and with bronchopulmonary infection in certain patient groups. However, no measurements of eosinophilic inflammation were performed in this study and patients with infections may have had asthma as well. A further limitation of this study was that the assay was based on antibodies against CLCP, which in subsequent studies has been conclusively shown to contain galectin-10 contaminated with lysophospholipase, a protein not specific to eosinophils [17]. Using antibodies specific to galectin-10 only, the present study extends the findings by Dor *et al* and demonstrates that galectin-10 is present only in diseases with an eosinophilic phenotype, and that galectin-10 quantity closely correlates with sputum eosinophil counts. Importantly, galectin-10 was found to lack a significant correlation with neutrophil percentages, strongly suggesting that it reflects eosinophil presence and would not be present in neutrophilic asthma. This is further evidenced by the absence of detectable amounts of galectin-10 in the healthy controls and interstitial pulmonary fibrosis patient.

Within the disease groups there was a large variation in eosinophil counts and galectin-10 measurements, particularly amongst the asthmatic patients. This could possibly be due to a constant change in the airway inflammatory profile over time in asthmatic patients, with fluctuations in eosinophil presence [31]. Though the asthmatic subtype (eosinophilic or non-eosinophilic) has been found to remain quite consistent in patients, it is the degree of inflammation within that subtype which changes, predominantly influenced by factors such as corticosteroids which reduces eosinophilic inflammation, or exposure to allergens which promote it [7], [9]. The range of galectin-10 measurements reflects the sensitivity of this protein to detect variations in eosinophilic inflammation. In contrast to eosinophil counts, sputum galectin-10 measurements does, however, have the potential to be further developed into a standard antibody-based diagnostic assay using raw or frozen sputum samples without laborious processing and manual counting.

The study's findings provide proof of concept that sputum galectin-10 could be an accurate alternative measurement of sputum eosinophils, either alone or in combination with other biomarkers. The current measurement of galectin-10 using the western blot method is semi-quantitative and future work should be directed towards developing a quantitative test for sputum galectin-10 which may further increase sensitivity of the assay, and improve its applicability to clinical practice. This will also allow future large scale clinical trials to evaluate galectin-10 as a surrogate biomarker to the current gold standard sputum eosinophil counts, and demonstrate its usefulness in asthma management.
